# Gold Nanostructures for Surface-Enhanced Raman Spectroscopy, Prepared by Electrodeposition in Porous Silicon

**DOI:** 10.3390/ma4040791

**Published:** 2011-04-14

**Authors:** Kazuhiro Fukami, Mohamed L. Chourou, Ryohei Miyagawa, Álvaro Muñoz Noval, Tetsuo Sakka, Miguel Manso-Silván, Raúl J. Martín-Palma, Yukio H. Ogata

**Affiliations:** 1 Institute of Advanced Energy, Kyoto University, Uji, Kyoto 611-0011, Japan; E-Mails: l-chourou@iae.kyoto-u.ac.jp (M.L.C.); r-miyagawa@iae.kyoto-u.ac.jp (R.M.); t-sakka@iae.kyoto-u.ac.jp (T.S.); y-ogata@iae.kyoto-u.ac.jp (Y.H.O.); 2 Department of Applied Physics, University Autonoma de Madrid, Carretera Colmenar Viejo, KM 15,500, 28049 Madrid, Spain; E-Mails: alvaro.munnoz@uam.es (A.M.N.); miguel.manso@uam.es (M.M.-S.); rauljose.martin@uam.es (R.J.M.-P.)

**Keywords:** gold, electrodeposition, porous silicon, Raman spectroscopy

## Abstract

Electrodeposition of gold into porous silicon was investigated. In the present study, porous silicon with ~100 nm in pore diameter, so-called medium-sized pores, was used as template electrode for gold electrodeposition. The growth behavior of gold deposits was studied by scanning electron microscope observation of the gold deposited porous silicon. Gold nanorod arrays with different rod lengths were prepared, and their surface-enhanced Raman scattering properties were investigated. We found that the absorption peak due to the surface plasmon resonance can be tuned by changing the length of the nanorods. The optimum length of the gold nanorods was ~600 nm for surface-enhanced Raman spectroscopy using a He–Ne laser. The reason why the optimum length of the gold nanorods was 600 nm was discussed by considering the relationship between the absorption peak of surface plasmon resonance and the wavelength of the incident laser for Raman scattering.

## 1. Introduction

Among biophysical and biochemical analyses, Raman spectroscopy plays an important role in the identification and structural characterization of molecules. Although Raman spectroscopy shows extremely low scattering cross sections as compared to fluorescence, the surface enhanced Raman scattering effect can provide cross sections with several orders of magnitude higher than normal Raman spectroscopy [1] and does not suffer from photobleaching effects [2]. Raman enhancement can be explained in terms of electromagnetic and chemical enhancement in the presence of metallic nanostructures such as silver, gold, copper and aluminum [3,4]. The most accredited theory predicts that the electromagnetic enhancement mechanism is largely responsible for the enhancement of Raman scattering. The magnitude of electromagnetic enhancement is determined by the localized optical properties of surface features at nanoscales. It is the intricate relationship between the surface structure and optical characteristics that determines the signal intensity. The oscillation of conduction electrons in metal nanoparticles can be driven by incident light. This electron oscillation, also known as the localized surface plasmon resonance, generates an electromagnetic field that is localized near the surface of a metal nanoparticle [5]. This large electromagnetic field induces a dipole in nearby molecules, thus enhancing Raman scattering from adsorbed molecules. The most commonly used substrates for surface-enhanced Raman spectroscopy are silver and gold colloids. However, the stability of the colloid solution and poor reproducibility due to aggregation are two major problems [6,7]. Another common substrate is a roughened metal electrode. Although these substrates are more stable than colloids, they are typically not so sensitive compared with the colloids [1,8]. Metal films evaporated on solid substrates or through shadow masks have also been found to exhibit controllable and reproducible surface-enhancement [9,10]. More recently nanorods of noble metals fabricated through various methods have been the focus of some reports [11,12,13].

Control in the shape and alignment of metal nanostructures is important for obtaining a high surface-enhancement. Utilization of porous template for the production of silver or gold nanostructures is a promising approach. Among many porous templates, porous silicon attracts attentions because porous silicon can be used as porous electrode without any special treatment. Although there are many publications studying the utilization of porous silicon, the number of studies on surface-enhanced Raman spectroscopy using porous silicon is still limited. This is because the deposition of silver and/or gold by electrochemical techniques is usually difficult due to the fast displacement deposition. Thus, almost all the studies on surface-enhanced Raman spectroscopy using porous silicon focused on the enhancement by electrochemically deposited metals on the top surface of porous silicon [14,15]. Only one paper reports the surface-enhanced Raman spectra from silver within porous silicon template, although the deposition was not carried out electrochemically [16]. Thus, it is usually difficult to play within porous silicon matrix. Recently, we have studied the electrodeposition of gold within mesoporous silicon. We found that the filling of gold nanoparticles and/or nanorods within mesoporous silicon was achieved by stabilization of Au ions via complexation with S_2_O_3_^2−^ and SO_3_^2−^, leading to the negative shift of the potential and the slowdown of the displacement deposition rate.

Herein, we report on a method for producing surface-enhanced Raman scattering-active gold nanorod arrays by electrodeposition using porous silicon. By changing the depth of porous silicon, we succeeded in the formation of gold nanorods with different lengths. We also found that a high surface-enhancement can be obtained by controlling the length of nanorods.

## 2. Results and Discussion

**Figure 1:**
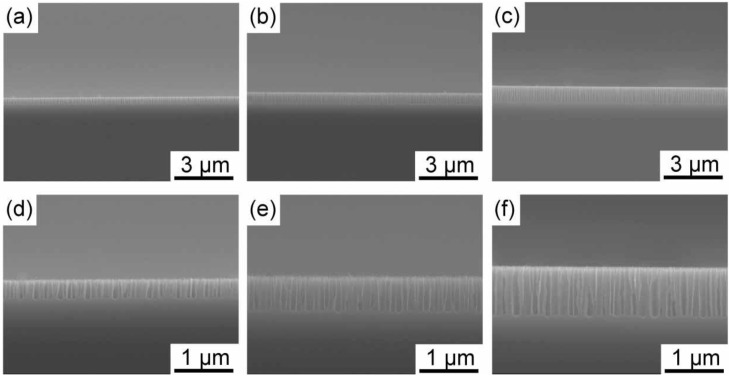
SEM images of medium-sized pores formed in n^+^-Si(100) at a current density of 44.6 mAcm^−2^ with different anodization times. The upper and lower rows show low and high magnification images. The anodization time was 14, 28, and 42 s for (**a**, **d**), (**b**, **e**) and (**c**, **f**), respectively.

It was revealed in a previous study that porous silicon with ~100 nm in diameter, which is so-called *medium-sized pores*, was formed using highly doped silicon in an HF solution containing an oxidizing agent [17]. In the present study, we utilized medium-sized pores as template for gold nanostructure formation. In Figure 1, images of medium-sized pores with different depths taken by a scanning electron microscope (SEM) are shown. As can be observed in the images after 14, 28 and 42 second anodizations, medium-sized pores grow almost linearly with time at the initial stage of the anodization. The pore depths reach ~300, ~600 and ~900 nm after 14, 28 and 42 second anodizations, respectively. The growth rate of the pores gradually decreased when the anodization time exceeded ~100 s. We decided to use three different medium-sized pores having different pore depths of 300, 600 and 900 nm as templates for gold electrodeposition.

**Figure 2:**
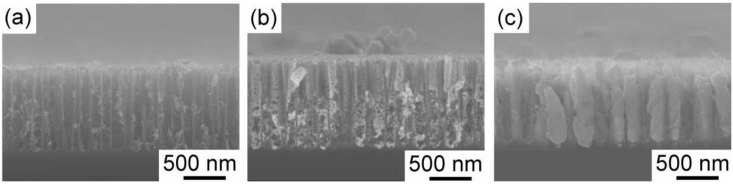
Time development of gold electrodeposition behavior within the medium-sized pores with 900 nm in depth. The gold electrodeposition was carried out at a current density of −12.7 mA cm^−2^ The electrodeposition times were 1, 4 and 6 h for (**a**), (**b**) and (**c**), respectively.

**Figure 3:**
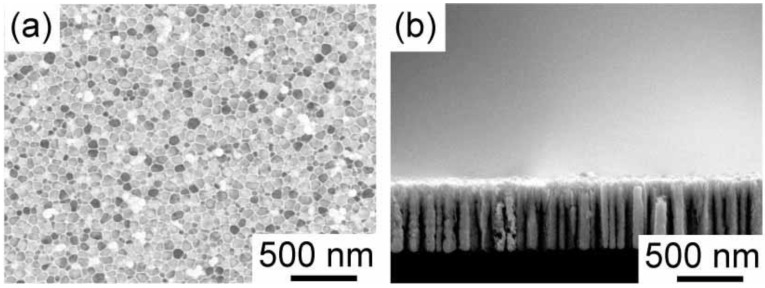
Gold-embedded porous silicon. The length of gold deposits is ~600 nm. The top and cross-sectional views are shown in (**a**) and (**b**), respectively.

**Figure 4:**
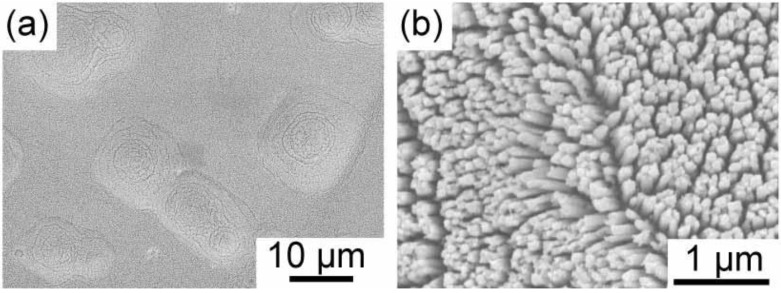
SEM images of a gold nanocarpet. The length of gold deposits is ~600 nm. The low and high magnification images are shown in (**a**) and (**b**), respectively. Additional 2 hour electrodeposition was performed from the condition in Figure 3 to form a thick film on the top of the porous layer. The porous silicon template was chemically dissolved by an alkaline solution (tetramethylammonium hydroide).

It is usually difficult to deposit gold within porous silicon. This is due to the fast rate of displacement deposition. In previous papers, we have reported that the displacement deposition of gold is suppressed by controlling the solution composition [18,19]. The reason why the displacement deposition is suppressed has been reported elsewhere [18]. Here, we also use the aqueous solution, which can inhibit the displacement deposition of gold, containing HAuCl_4_, Na_2_SO_3_ and Na_2_S_2_O_3_. Figure 2 shows the time development of gold electrodeposition within the porous silicon with 900 nm in depth. After one hour electrodeposition, the nuclei of gold are deposited on the pore wall. They randomly distribute on the wall. The nuclei grow along the pore wall, and then tubular gold deposits start to grow from the pore opening to the bottom as shown in Figure 2b. Finally, the tubular deposition reaches to the bottom, and the tubes starts to thicken. If the electrodeposition time is precisely controlled, we obtain gold nanorods deposited into medium-sized pores. An example is shown in Figure 3. The porous silicon with 600 nm in depth is filled with gold, and overdeposition on the top surface does not occur. Judging from the top view, the pores are completely filled, and the gold deposits are not tubular. Additional two hour electrodeposition after the deposition reaches to the pore opening leads to filmy growth on the top surface of the porous silicon. In Figure 4, SEM images of the gold deposit after the dissolution of porous silicon template by the chemical etching with tetramethylammonium hydroxide are shown. A gold nanorod array is obtained, and they are well aligned due to the original pore alignment (Figure 4b). In the low magnification image (Figure 4a), some dimples are observed. These dimples may be formed due to residual glue of the double-stick tape. The gold nanorod array was fixed to a glass plate with a double-stick tape, so that the array can be used for Raman spectroscopy after stabilizing the structure mechanically. Hereafter, we will describe the gold nanorod arrays (Figure 4) as *gold nanocarpets*, while the gold nanorods within the porous silicon template (Figure 3) as *gold-embedded porous silicon*.

**Figure 5:**
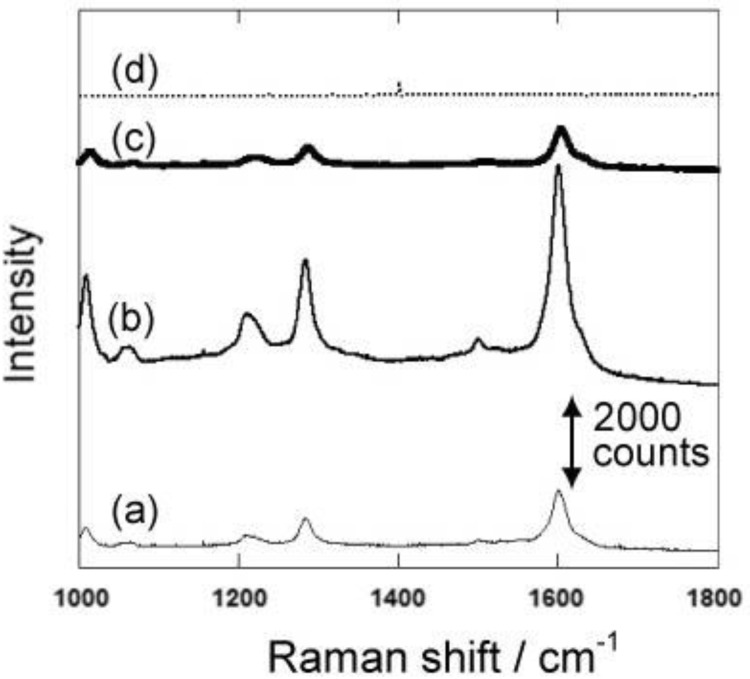
Raman Spectra of 4,4’-bipyridine using the gold nanocarpets with different nanorod lengths. The length of gold nanorods is 300, 600 and 900 nm for the spectra shown as (**a**), (**b**) and (**c**), respectively. The spectrum indicated as (**d**) is obtained with a mirrored gold film.

**Figure 6:**
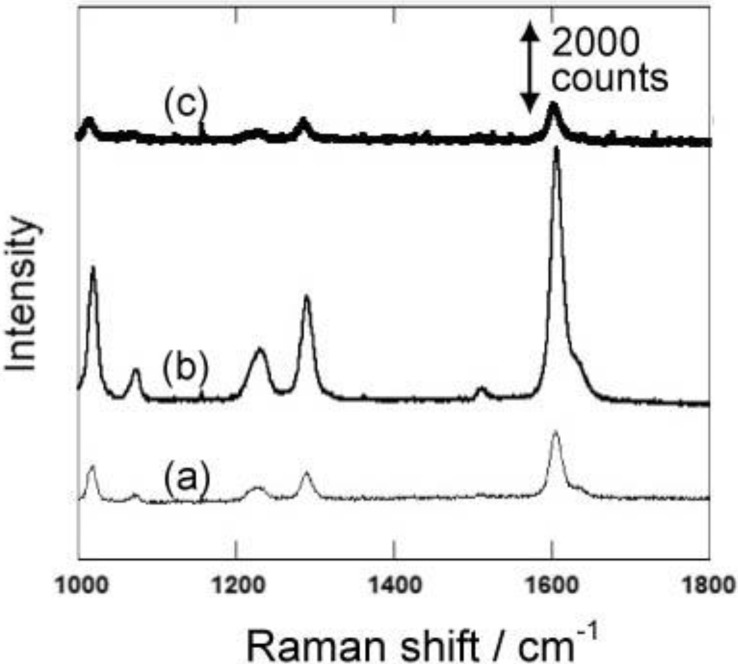
Raman Spectra of 4,4’-bipyridine using the gold-embedded porous silicon substrates with different nanorod lengths (different depths). The length of the gold nanorods in porous silicon is 300, 600 and 900 nm for (**a**), (**b**) and (**c**), respectively.

Gold nanocarpets with different lengths can be easily prepared by changing the depths of the medium-sized pores. We prepared gold nanocarpets with three different lengths, namely 300, 600 and 900 nm. To obtain the nanocarpets, the porous silicon matrices were completely dissolved by chemical etching. Using the three types of gold nanocarpets, the surface-enhanced Raman spectroscopy of 4,4’-bipyridine was conducted. Figure 5 shows the surface-enhanced Raman spectra of 4,4’-bipyridine. The highest Raman intensity is obtained by the gold nanocarpet with 600 nm in length. Although the surface area for the adsorption of the target molecule is much larger with 900 nm in length than the 600 nm one, Raman intensity is higher in the 600 nm carpet than in the 900 nm one. These results suggest that the obtained Raman intensity is not simply explained by the amount of adsorption on the gold nanocarpet. In order to support this discussion, we also measured Raman spectra with gold-embedded porous silicon samples with 300, 600 and 900 nm in depth (Figure 6). The Raman intensities decrease in all the samples due to the decrease of the area for the adsorption. However, it should be mentioned that the highest surface-enhanced Raman intensity is again obtained with 600 nm length although the accessible gold surface is limited only to the tips of the gold nanorods. Thus, these spectra also strongly suggest that the amount of adsorption is not the main factor to understand the Raman intensity.

**Figure 7:**
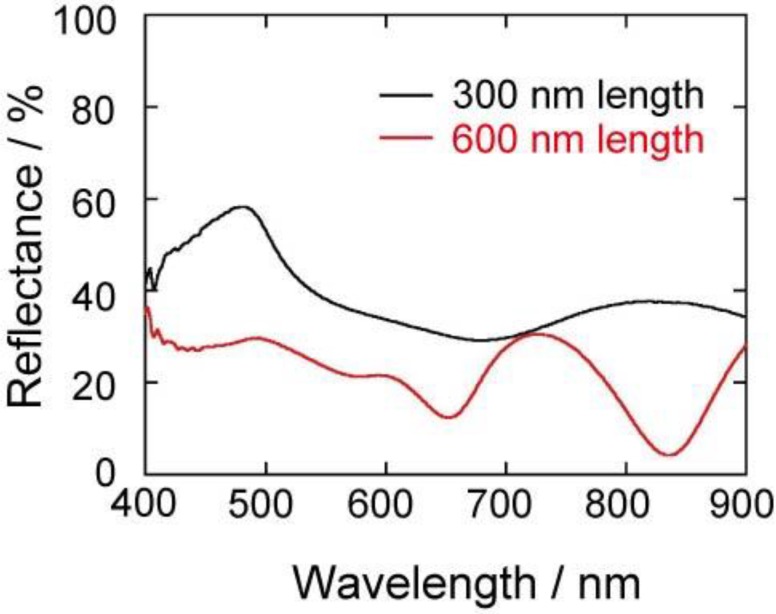
Visible absorption spectra of the gold nanocarpets with nanorods of 300 and 600 nm in length. The black and red curves are the spectra of the nanocarpets with 300 and 600 nm in rod length, respectively.

The enhancement may originate from the surface-plasmon properties of the gold structures. In order to understand the surface-plasmon properties, the visible absorption spectra of the gold nanocarpets were measured. The absorption spectra were measured with a reflection technique using an optical microscope. The reflectance of a compactly and smoothly deposited gold film was used as the reference spectrum. In the measurement of plasmon absorption, we selected a relatively smooth part of the carpet and avoided the measurement of the absorption spectra on the dimples on which residual glue of the double-stick tape may remain. Figure 7 shows the visible absorption spectra of the gold nanocarpets with 300 and 600 nm in length. We do not show the spectrum of the 900 nm carpet because the reflectance of the sample was quite weak and unclear, probably due to the light trapping by the highly porous surface of the carpet. In the spectrum of 600 nm carpet, two strong absorption peaks are clearly observed at 640 and 830 nm, and a shoulder is observed at ~530 nm. The peaks at 640 and 830 nm originate from the latitudinal and longitudinal modes of surface-plasmon. It is known that gold nanoparticles show absorption at ~520 nm, although it depends on the size of nanoparticles. In our gold carpet, the surface of the gold nanorods is not smooth, but rough, as if the rods are modified with gold nanoparticles. Therefore, the origin of the third weak absorption at ~530 nm might be due to the roughened surface of the nanorods. Then, let us see the spectrum obtained with the 300 nm nanocarpet. A very wide peak exists from 500 to 800 nm, and the spectrum is not symmetrical. Again, a shoulder is observed at ~540 nm. This might be from the same origin as the ~520 nm absorption observed with the 600 nm carpet. In this spectrum, we cannot observe two clear peaks as observed in the 600 nm carpet. This may be because the peaks of longitudinal and latitudinal modes exist very closely due to the short length of the nanorods. By comparing the two spectra, we think that the higher enhancement of the Raman intensity with the 600 nm nanocarpet and the rod-embedded porous silicon is caused by an effective excitation of surface-plasmon with a He-Ne laser (λ: 633 nm). The nanocarpet with 600 nm rod length has surface-plasmon absorption at the wavelength of the incident laser. This overlap induces the higher surface-enhancement. Thus, the optimum length of the gold nanorod should be different, and it depends on the type of incident laser.

It should be noted that the Raman intensity is homogeneous on the whole carpet (~1 cm in diameter) as reported in our previous paper [20]. In the paper, five different sites were randomly selected, and the Raman intensity of 4-4’ bipyridine was measured. The Raman intensities were almost homogeneous. Five samples were analyzed, and then sample to sample variation was not distinguishable. These results suggest that the nanocarpet does not have hot spots, at which extremely high enhancement is expected. If the nanocarpet has hot spots, plasmon absorption and the incident laser do not need to be in resonance. Because the nanocarpet does not have hot spots, the absorption and the wavelength of the incident laser should be in resonance to induce relatively high enhancement on the nanocarpet. Enhancement factor also seems to be important to understand the enhancement behavior. The enhancement factor of this nanocarpet can be evaluated as reported in [21]. However, the amounts of the target molecules adsorbed on the nanorods and the precise size of analyzing area are necessary to evaluate the enhancement factor. We are currently trying to evaluate the parameters to obtain the enhancement factor.

## 3. Experimental Section

A highly doped *n*-type (n^+^) silicon (100) with a resistivity of 0.010–0.018 Ωcm was used as a substrate. The substrate was rinsed in ultra pure water and acetone under ultrasonic irradiation for 5 minutes. Before the formation of porous silicon, the native oxide layer was removed in 5 wt% HF aqueous solution. The preparation of porous silicon was carried out under current controlled condition in an HF aqueous solution written below with a two-electrode cell. The Si substrate (0.71 cm^2^) and a Pt rod (1 mmФ × 2 cm ) were used as the working and counter electrodes, respectively. The HF solution was 6 wt% HF (Sterachemifa, 47 wt%) + 3000 ppm of NCW-1001 (Wako chemicals) + 8 mM KMnO_4_ (Wako chemicals, analytical grade). The anodizing current density was 49.3 mAcm^−2^.

The electrodeposition was carried out in the dark and at room temperature under a cathodic current density of 14 µAcm^−2^. The solution for electrodeposition was 0.01 M HAuCl_4_ (Nacalai Tesque) + 0.42 M Na_2_S_2_O_3_ + 0.42 M Na_2_SO_3_. To obtain nanorods with ~300, ~600 and ~900 nm length, the electrodeposition time was adjusted to 2.5, 3.5 and 5.5 h, respectively. After gold electrodeposition, the Si template was dissolved by alkaline etching in a 25 wt% tetramethyl ammonium hydroxide aqueous solution (Aldrich) at 363 K.

To investigate the activity of surface-enhancement, a droplet of 100 µM 4-4’bypiridine was put on the nanorod structure for 1 h. After drying the substrate with air, Raman scattering was measured by a micro-Raman spectrometer (HORIBA Jobin Yvon Inc., Labram010) in air using a He–Ne laser (λ: 633 nm, 6.4 mW) as an incident laser. Raman spectra were obtained by a single scan with 300 s exposure.

The samples were observed by a field emission-type scanning electron microscope (JEOL FE-6500, SEM).

The visible absorption spectra were measured by an absorption spectrometer combined with an optical microscope (Nikon, ECLIPSE LV100D). The absorption measurements were performed in air.

## 4. Conclusions

Gold electrodeposition within medium-sized pores formed in n^+^-silicon was performed, and then we obtained gold nanocarpets and gold-embedded porous silicon substrates. The surface-enhanced Raman spectroscopy of 4,4’-bipyridine showed an interesting feature depending on the nanorod length. The highest intensity was obtained when using the 600 nm nanorods regardless of the morphologies with and without porous silicon substrate, namely the gold nanocarpet and gold-embedded porous silicon. The enhancement of the Raman intensity was explained by the overlap of the plasmon resonance peak and the incident laser. Such gold nanostructures can be useful as substrates for sensing and photochemical reactions.
